# Development of a Family-Centered Communication Tool for Kidney Health in Premature Infants: Qualitative Focus Group Study Using Human-Centered Design Methodology

**DOI:** 10.2196/45316

**Published:** 2023-07-10

**Authors:** Michelle C Starr, Samantha Wallace, Courtney Moore, Brandon Cockrum, Bridget Hawryluk, Aaron Carroll, William Bennett

**Affiliations:** 1 Division of Pediatric Nephrology Department of Pediatrics Indiana University School of Medicine Indianapolis, IN United States; 2 Center for Pediatric and Adolescent Comparative Effectiveness Research Indiana University School of Medicine Indianapolis, IN United States; 3 Research Jam Indiana Clinical and Translational Sciences Institute Indianapolis, IN United States

**Keywords:** qualitative research, patient-reported outcomes, neonates, chronic kidney disease, human-centered design, acute kidney injury, kidney health

## Abstract

**Background:**

Premature infants are at increased risk of kidney-related complications, including acute kidney injury (AKI) and chronic kidney disease (CKD). The risk of CKD in prematurely born infants is underrecognized by health care teams and caregivers. Understanding how to communicate the risk of CKD to caregivers is essential for longitudinal clinical follow-up and adherence.

**Objective:**

This study aimed to determine family caregiver attitudes toward kidney health and risk communication during a neonatal intensive care admission. We also sought to understand caregiver preferences for the communication of information surrounding the risk of CKD in premature infants.

**Methods:**

We augmented standard qualitative group sessions with human-centered design methods to assess parent preferences and clinician perspectives. Caregivers recruited had a prematurely born child who spent time in the neonatal intensive care unit at Riley Hospital for Children in Indianapolis, Indiana, and experienced AKI or another kidney complication, which put them at risk for future CKD. We used a variety of specific design methods in these sessions, including card sorting, projective methods, experience mapping, and constructive methods.

**Results:**

A total of 7 clinicians and 8 caregivers participated in 3 group sessions. Caregivers and clinicians readily acknowledged barriers to and drivers of long-term kidney monitoring as well as opportunities for communication of the risk of long-term kidney disease. Caregivers’ primary concerns were for both the type and depth of information conveyed as well as the time at which it was communicated. Participants emphasized the importance of collaboration between the hospital care team and the primary care provider. Participant input was synthesized into several prototype concepts and, ultimately, into a rough prototype of a website and an informational flyer.

**Conclusions:**

Caregivers of premature infants are open to communication about kidney health during their neonatal admission. The next phase of this work will translate caregivers’ preferences into family-centered communication tools and test their efficacy in the neonatal intensive care unit.

## Introduction

Premature infants are at high risk of kidney-related complications, including acute kidney injury (AKI) and chronic kidney disease (CKD) [1]. AKI is common in premature infants, occurring in between 20% and 40% of infants, depending on the patient population studied [2,3]. Premature infants with AKI have higher rates of mortality and longer hospital stays [2,3]. The risk of kidney-related complications in premature infants does not disappear after the neonatal admission. Studies in prematurely born children show a 4-fold increase in CKD during childhood and adolescence [4-9]. While likely multifactorial, one explanation for this increased CKD risk is that premature infants are born with a decreased number of nephrons due to their early delivery [10]. Furthermore, the extrauterine environment (including the use of nephrotoxic medications and perinatal stressors) may not be amenable to proper nephron development [11]. Even children with normal kidney function but a history of AKI have a 10 times higher risk of developing kidney failure before the age of 40 years [12,13]. Thus, as more critically ill infants survive and live into adulthood, the impact of kidney health on premature infants is a significant long-term concern.

Communication surrounding kidney health to families, specifically focusing on the risk of CKD, is essential in empowering families and ensuring longitudinal clinical follow-up and monitoring. Studies show that kidney health, including the diagnosis of AKI and the risk of CKD in prematurely born infants, is underrecognized by health care teams [14,15]. While there are no established best practices for communication in the neonatal intensive care unit (NICU), families of premature infants report a desire for direct and concise communication during their NICU stay, focusing on the most urgent or immediate clinical concerns [16,17].

There have been no studies which evaluate kidney-specific health communication with families. The purpose of this study was to fill this gap by evaluating caregiver attitudes toward kidney health and CKD risk communication as well as caregiver preferences for communication of information surrounding the risk of CKD. This study serves as the first step in the development of a family-centered tool to improve communication about kidney health in premature infants.

## Methods

### Overall Approach

In collaboration with Research Jam and the Indiana Clinical and Translational Science Institute’s Patient Engagement Core, we conducted 2 phases of group sessions using qualitative focus group methodology augmented by human-centered design methods (Multimedia Appendix 1). Human-centered design, which is increasingly used within health care, is an iterative design process where stakeholders most closely affected by the problem or solution are engaged in developing the solution [18,19].

Sessions were facilitated by 4 research specialists using human-centered design research methods. Sessions were held virtually through Zoom (Zoom Video Communications), lasted approximately 120 minutes each, and were recorded and transcribed for analysis. All sessions used activities to engage clinicians and caregivers to better understand caregiver perspectives on communication surrounding kidney disease as a first step in the co-design of a kidney disease communication tool [20,21]. Activities were open-ended, allowing for a wide range of responses to minimize bias and for families to be as open and truthful as possible about their preferences. Sessions began with warm-up activities to encourage participation and collaboration [22]. We then used specific generative activities (eg, empathy mapping, detailed below) designed to encourage study participants to express their thoughts and feelings and constructive methods to help with concept development [21]. All sessions used Miro Whiteboard (Miro) [23], a collaborative whiteboard platform which the group facilitator used to document and visualize responses in real-time for the group.

### Recruitment, Subjects, and Study Setting

Stakeholders included clinicians and caregivers. Clinicians were from across the United States and cared for prematurely born children who spent time in the NICU. This included physicians, nurses, and nurse practitioners trained in pediatric nephrology, general pediatrics, and perinatal and neonatal medicine, all of whom were approached and recruited by the principal investigator.

Caregivers were recruited who had prematurely born children who spent time in the NICU at Riley Hospital for Children in Indianapolis, Indiana. Caregivers were approached for enrollment in this qualitative study if their child was: (1) born prematurely and admitted to the NICU during their infancy; or (2) experienced AKI or another kidney complication (such as a slow to normalize serum creatinine), which put them at risk for future CKD. Caregivers were eligible for this study if their child was between ages 2 and 25 years old, if they agreed to participate in the web-based session, and if they had no diagnosed cognitive disabilities.

Recruitment was conducted by phone as well as in the outpatient pediatric nephrology clinic at Riley Hospital for Children, part of Indiana University Health, in Indianapolis, Indiana. Permission to approach the caregiver was obtained from the nephrologist of record to ensure the child did not have any medical treatments or conditions that could deter participation in the session. Informed consent was obtained from each study participant. Study participants were given a US $100 Amazon gift card for their engagement.

### Exploring and Co-Design

We held 2 virtual sessions that were identical in purpose and methods but engaged different stakeholder groups. The first session included clinicians, and the second session included caregivers (Multimedia Appendix 1).

Specific activities included during the exploring and co-design sessions included:

#### Empathy Mapping

Empathy mapping is a generative method in which stakeholders are asked to intentionally speak about different aspects of an experience (thinking and feeling, hearing, seeing, and saying and doing) [24]. Stakeholders (clinicians and caregivers) were asked to address each of these areas based on the following prompt: “After their child has received life-saving drugs in the NICU, parents are told that their child will need lifelong kidney monitoring. Help us understand this conversation.” To understand the context, stakeholders were also asked to describe where, when, and how this conversation took place. In addition, stakeholders were asked about the barriers to and drivers of lifelong kidney monitoring.

#### Co-Design

Co-design refers to the practice of guiding caregiver and clinician co-designers in the design development process [25]. The following co-design methods were used:

1. *Concept generator:* we created a worksheet in Miro to help caregiver and clinician co-designers diverge and converge on the function and form of a potential tool. It included the following instructions:

We need to develop a tool to help patients and their families overcome their barriers to long-term kidney monitoring. Let’s think creatively about what that tool could be.

1. *Prototyping:* creating a rough version of a solution (a prototype) gave designers and caregiver and clinician co-designers the opportunity to make rough ideas tangible to quickly gain feedback and make iterations. Prototypes displayed the approximates of the solution or part of the solution [26]. How the prototype looked at this stage was less important than the conversation about why features were included and what problems each feature solved. We created a worksheet in Miro to help stakeholders create their prototypes.
2. *Rose, Thorn, Bud:* Rose, Thorn, Bud was a reflective activity used during the session to get stakeholders to intentionally think about each prototype and provide feedback [27]. As a group, stakeholders focused on 1 prototype at a time and then shared 3 things: something that they thought was working well (a rose), something that presented a challenge (a thorn), and something that represented an opportunity or idea with potential (a bud).

### Analysis of Exploring and Co-Design Sessions

Data (including the developed products, notes, and transcripts) from the sessions were analyzed using John Kolko’s methods of analysis and synthesis, using a creative process to connect research insights with design patterns to generate well-grounded design ideas [28]. These data were grouped by affinity or similarity of content, with each group given a heading to summarize its content. The resulting affinity diagrams spatially organized the data into groups based on similarity of content and represented the full picture of the data organized by theme [29]. Next, an analysis team created visual models of the themes and how they were interrelated [28].

Models included a refined empathy map, a communication opportunities map, and a grouping of “must have,” “can’t have,” and “nice to have” features for the communication tool. During model-building, a total of 2 “must have” and “nice to have” continuums were created. Each of the educational content and bonus feature items were placed on their respective continuums as determined by participants. Discussion points collected during the sessions were placed below their related item in the continuum.

### Prototype Development

The research team then moved to prototype development, which looked at the outcomes of analysis (“what is”) to build solutions for the future (“what could be”) using the following synthesis methods:

#### Brainstorming Potential Challenges to Solve

To diverge further on what the solution could be, the team identified underlying challenges within the main objective. Asking “how might we...” allowed the research team to think beyond first instinct responses and use a divergent mindset to come up with many potential ideas for solutions. The research team then converged on the challenges that best fit the objective and what was learned from the analysis.

#### Brainstorming Potential Solutions for Selected Challenges

The research team asked one “how might we...” question at a time and listed as many solutions as they could. The research team used a divergent mindset, limited judgement, and focused on quantity over quality. Thinking broadly allowed for the generation of out-of-the box solutions that could be examined for valuable elements that could be implemented into a final tool.

#### SCAMPER Method to Diverge on Additional Solutions

To further diverge, the research team used the SCAMPER method to create new solutions by manipulating already-stated solutions [30]:

- Substitute: what could you substitute or change?
- Combine: could two or more ideas or pieces be combined into something else?
- Adapt: what could be tweaked to improve the solution?
- Modify: could some solutions be changed to be improved?
- Put to another use: could solutions apply to another use?
- Eliminate: what could we take away from these solutions to improve them?
- Reverse: would rearranging elements improve solutions?

The research team used each of these prompts to create new solutions based on the existing process or solutions from the previous step. Following the divergent stage, the research team reviewed the list of solution ideas and voted for those they thought were the most appropriate and interesting.

#### Prototyping

The research team individually created prototypes of the tool inspired by the converged list of solution ideas, allowing the research team to explore additional ideas that could be included in the final tool. Refined prototypes were then used to get feedback from the stakeholders.

### Prototype Refinement

We held 1 virtual session with a subgroup of clinicians and caregivers by Zoom to evaluate the prototypes developed using Miro.

1. *Sorting to Prioritize Prototype Content and Features:* Study participants were presented with a list of potential educational and informational elements identified as either “must have” or “nice to have” by the research team. Study participants were then asked to discuss and sort each of these into one of the two categories themselves. This same approach was taken with a list of bonus features the tool could include. This activity allowed for potential elements to be categorized based on the perspectives of the stakeholders, not just the research team.
2. *Prototype Feedback using Rose, Thorn, Bud:* Study participants were shown 2 prototypes. Each prototype had 3 main elements: information and education, bonus help, and appointment reminders. Prototype A focused primarily on digital solutions, while prototype B focused on analog solutions. The research team presented both prototypes to the study participants, asked for clarifying questions, then worked through the same Rose, Thorn, Bud activity used in phase 1 to get feedback for each prototype. This activity helped the research team understand elements of the prototypes that stakeholders liked and disliked.
3. *Frankenstein Prototypes:* With knowledge and opinions about what should go into the tool, study participants were asked to build new prototypes using their favorite elements from prototypes A and B. With the ability to mix, match, and create new elements, the research team could see what the participants prioritized.

### Analysis of Prototype Refinement

We used affinity diagramming to group the feedback provided during Rose, Thorn, Bud. Through discussion within the research team, feedback from participants was arranged into groups and given thematic headings. These headings were used to identify key elements that study participants liked, did not like, and saw as having potential in the prototypes presented to them, allowing the research team to make final decisions about how to refine the prototypes. The research team then reviewed each item on the continuum and made decisions about what should be included in the final communication tool. Decisions were made based on feasibility and how well the item would address the original objective.

### Ethics Approval

This study was approved by Indiana University’s institutional review board (protocol #11958), by whom it was deemed minimally risky.

## Results

### Participants

The exploring and co-design sessions included 15 participants (7 clinicians and 8 parents), while the prototype refinement session included 10 participants (6 clinicians and 4 caregivers). We approached 20 clinicians (7/20, 35% participation rate) and 32 caregivers (8/32, 25% participation rate; Multimedia Appendix 1). All the patients represented by caregivers in this study were discharged from the hospital and were currently seeing pediatric nephrology for monitoring of kidney health or management of CKD. See Table 1 for demographic characteristics for the study participants in the exploring and co-design sessions.

**Table 1:** Demographic characteristics for study participants in the exploring and co-design sessions.

			Clinicians, N=7		Caregivers, N=8
**Gender, n (%)**					
	Female	4 (57)		6 (75)	
	Male	3 (43)		2 (25)	
**Age (in years), n (%)**					
	21-44	6(86)		7 (88)	
	45-64	1 (14)		1 (12)	
	65 and older	0		0	
**Race, n (%)**					
	Asian	1 (14)		1 (12)	
	Black or African American	1 (14)		1 (12)	
	White	5 (72)		6 (75)	
**Ethnicity, n (%)**					
	Hispanic or Latino	1 (14)		2 (25)	
	Not Hispanic or Latino	6 (86)		6 (75)	
**Clinical subspecialty**					
	General pediatrics	1 (14)		N/A	
	Neonatal and perinatal medicine	2 (28)		N/A^a^	
	Pediatric nephrology	4 (58)		N/A	
Child’s current age (in years), mean (SD)			N/A		6 (4)

^a^N/A: not applicable.

### Caregiver Experience

The caregiver experience began with their infant’s admission to the NICU. Sometimes, caregivers and clinicians expected that an infant would require immediate medical intervention after birth, while other times it was unexpected. Either way, infants required medical care in the NICU, with their caregiver as the primary decision maker. About this moment, one participant said (paraphrased): “I sat and looked at this perfect baby and they’re telling us she has all these challenges.” During the course of medical care, parents were often involved in difficult decisions or treatment decisions, such as clinicians recommending the use of life-saving medications and treatments that could harm their kidneys (eg, nephrotoxic medications, surgery, and other interventions; Multimedia Appendix 2).

In addition to making decisions critical to their infant’s care, the physical location of the decision placed additional stress on caregivers. Frequently, discussions between caregivers and clinicians occurred in the NICU, sometimes privately but often in the proximity of other patients and passersby. Caregivers described an overwhelming scene with many new sights and sounds, hopes and fears, high and low emotional points, and advanced levels of stress and fatigue. As doctors presented the treatment options and the implications of those options, caregivers found it easy to lose focus and not remember all the details of the conversation. They may or may not remember being informed that the child would need lifelong kidney monitoring due to potential kidney damage from life-saving treatments (Multimedia Appendix 2).

### Barriers and Drivers to Monitoring

**Figure 1:**
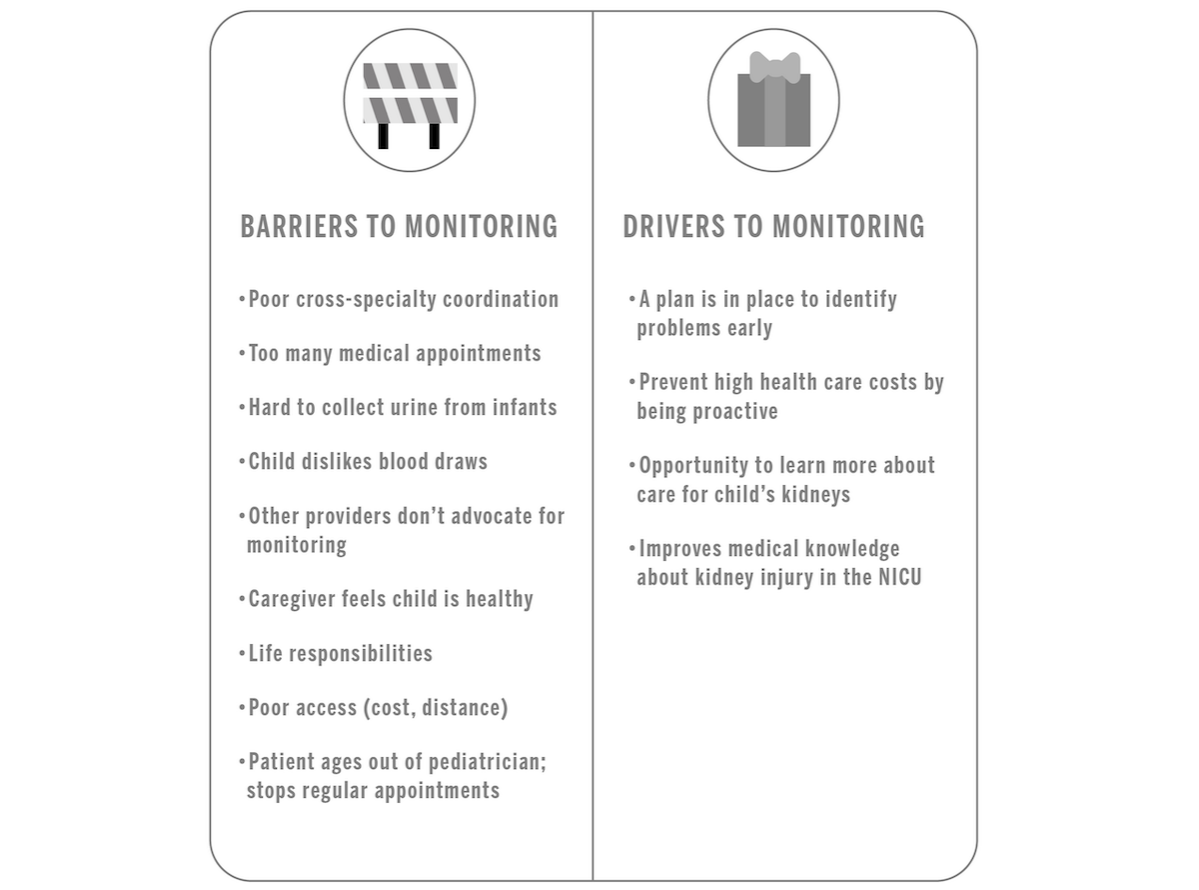
Barriers and drivers to long-term kidney monitoring for children after the neonatal intensive care unit (NICU).

Some caregivers recalled that clinicians suggested the need for kidney monitoring at the time of discharge. Both caregivers and clinicians readily acknowledged barriers to and drivers of long-term kidney monitoring. Caregivers shared that many of their pediatricians and other health care clinicians agreed with or reinforced the need to monitor the patient’s kidneys; however, at least one caregiver was told that it was not necessary by their pediatrician. Adherence to kidney health monitoring, in addition to other treatments that may be required following their NICU admission, posed more immediate challenges, such as the difficulty of their young child tolerating a blood draw or urine collection. Caregivers weighed these barriers versus the drivers of early identification of kidney problems, improved care for their child, and saving money over time. Barriers to and drivers of long-term kidney monitoring are summarized in Figure 1 and Multimedia Appendix 2.

### Communication Considerations and Opportunities

**Figure 2:**
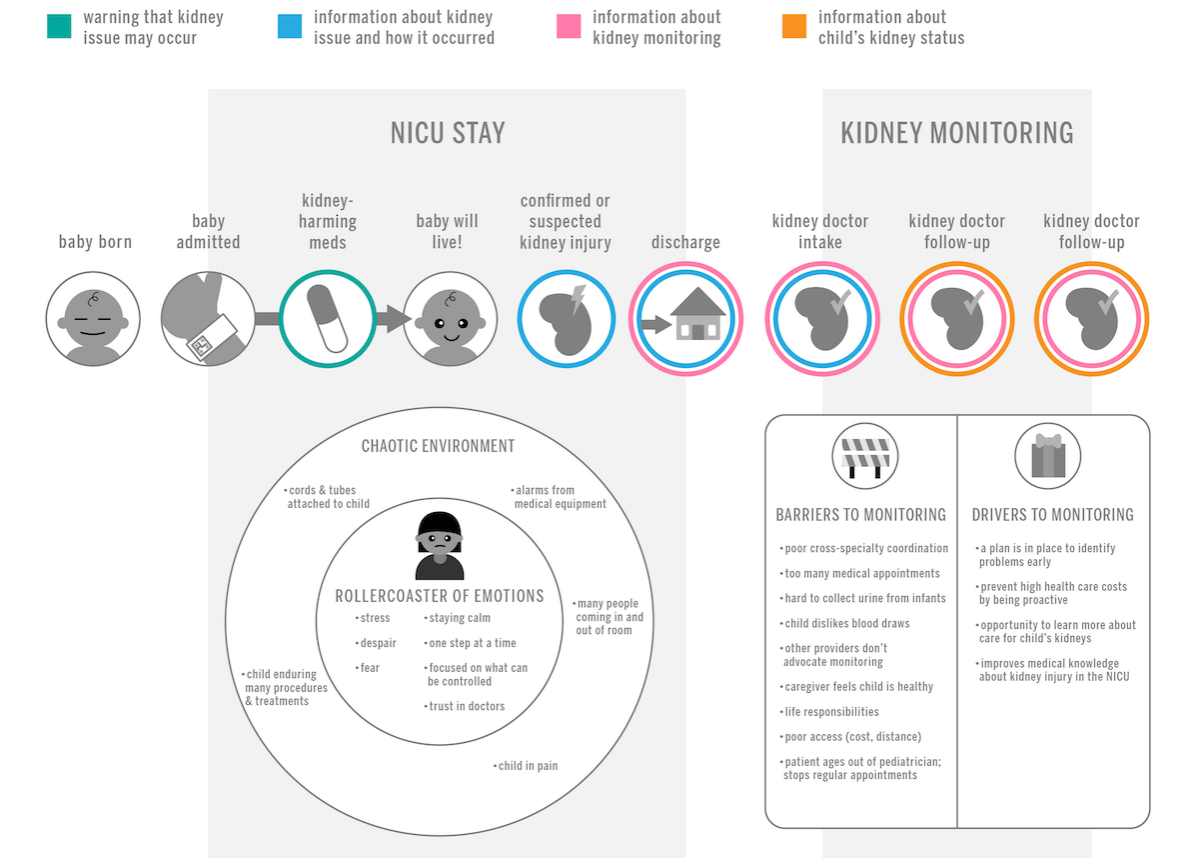
Communication opportunities for kidney monitoring during and after neonatal intensive care unit (NICU) stay.

Stakeholders reported positive and negative aspects of the communication of medical information, both generally and about the implications of kidney injury specifically (Multimedia Appendix 2). Caregivers noted that due to the stressors experienced by caregivers and the challenges of learning and memory retention in the NICU, clinicians should offer information about kidney health and long-term kidney monitoring at multiple points throughout the NICU admission, including at the time of administering medications or therapies that may contribute to kidney injury, at discharge as part of the discussion of follow-up care needed, and at follow-up appointments. Figure 2 shows a model of caregiver experience with communication opportunities identified.

### Contents and Features

Study participants sorted educational content and potential features into “must have” and “nice to have” categories. Each of the educational contents was placed on their respective continuums based on where it was placed by study participants (Table 2). For example, “questions to ask clinicians” was placed in the “must have” section of the education and information continuum because both groups sorted it as a “must have,” while “NICU guide” was placed both in the “must have” section and the “nice to have” section because 1 group sorted it as a “must have” item and the other as a “nice to have item.”

**Table 2:** “Must have,” “nice to have,” and “should not have” components determined by caregivers and clinicians.

	Must have	Nice to have	Should not have
Educating caregivers and patients	Kidney condition treatment and monitoring Tips for lifelong monitoring Real-world experiences Benefits of monitoring Appropriate tone Presented in simple terms Use visuals	Real-world implications of kidney injury Guide to the NICU^a^ and kidney care How to advocate for your child Clinic visit guide Laboratory testing guide Questions to ask clinician Help with blood work and how to collect urine Gamify education Use videos Intentionally build clinic or follow-up retention	Make caregivers feel guilty about their child’s kidney disease risk
Enhances communication	—^b^	Communication between patient and clinician Questions and answers space, or frequently asked questions NICU doctor livestream video Communication between clinicians Share laboratory results	Avoid increasing work burden of clinicians
Make scheduling appointments easier	—	Scheduling Help stacking and coordinating appointments to one visit Appointment tracking and reminders	—
Track and sense make of laboratory results for caregivers	Explain and interpret laboratory results	Longitudinal tracking of laboratory results Alert for concerning laboratory results	—
Identify treating health care team for caregivers	—	Staff profiles and list	—
Support for caregivers	—	Offer community and support	—
Help caregivers	—	Note taking, keeping resources together Longitudinal life of tool Custom to patient	—
Access information outside of the stressful NICU setting	App Website Printed materials	Translate into different languages Content on tablet at hospital	—

^a^NICU: neonatal intensive care unit.

^b^N/A: not applicable.

The research team then reviewed each item on the continuum and made decisions about what should be included in the final tool. The research team also discussed which of the items from the middle 2 sections should be included. The research team reviewed the bonus features and decided which of these to include in the final tool. Decisions were made based on how well the item would address the original objective.

### Prototypes and Feedback

The research team created 2 prototype web pages to illustrate what a final tool might look like (Multimedia Appendix 3). For example, the home page included information about poor kidney development, potential kidney injury in the NICU, and how this may lead to the need for long-term kidney monitoring. It also contained a still from a video that might exist where a clinician explains NICU kidney injuries. The home page acts as the basic information for caregivers, while the rest of the site offers additional details. The menu items included: “about kidney monitoring,” “common kidney tests,” “talking with your child’s doctor,” and “caregiver support.” Caregivers and clinicians reviewing the prototypes were supportive of the categories of information and content provided. They also appreciated the overall design of the prototype webpage. In general, they wanted caregiver stories with diverse people and languages, as well as more detailed information and research.

## Discussion

We conducted a qualitative study examining caregiver attitudes and preferences toward the communication of kidney health by clinicians in the NICU setting. Our results suggest opportunities for improving communication about the risk of long-term kidney disease between caregivers and clinicians. Caregivers’ primary concerns were the type and depth of information conveyed and the time at which it was communicated. Both caregivers and clinicians emphasized the importance of collaboration between the NICU team and the primary care provider to ensure they were on the same page about the necessity of kidney monitoring.

This study represents the first attempt, to our knowledge, to develop a set of clear approaches to communicating kidney health and the risk of CKD in the NICU. Our findings are in concert with a recently published survey of caregivers with infants diagnosed with necrotizing enterocolitis during their NICU admission [17]. Both studies found that caregivers desired accurate and timely information to inform care and improve communication. Furthermore, other studies suggest that information gathering is an important coping mechanism for stress while their child is in the NICU [31]. Education-based programs have additional benefits for caregivers, including improved parental mental health outcomes, stronger beliefs in their parental role, and increased parental engagement [32]. The timely and family-centered provision of information and education is an essential aspect of family-centered care, which has increased parent engagement and satisfaction as it has become more widely used in NICUs over the last decade [33].

One challenge in neonatal kidney health clinical care and research studies is the low rate of kidney-specific follow-up for infants [34,35]. Studies suggest that, while multifactorial, contributing factors include poor provider and caregiver awareness of the risk of long-term kidney disease, a lack of family communication, and a perceived inability to change the course of disease with care [36]. Furthermore, siloing of care and electronic health care records which do not follow patients from health encounter to health encounter limit the ability of caregivers and clinicians to carry health information with them throughout the medical system. The development of improved communication with caregivers during and after their NICU stay is paramount to improving not only clinical care but also research studies of long-term kidney health, which are often stymied by poor retention. Our approach to kidney health communication was developed not by expert consensus of clinicians, as is often the case in similar studies, but by directly engaging with caregivers who have had infants admitted to the NICU who are at risk of long-term kidney disease. We believe this will result in a far more effective communication strategy that is more acceptable to families and increases the efficacy of subsequent follow-up.

There are several important limitations to this study. First, owing to the relatively small sample size and narrowness of the study population (eg, caregivers of infants at risk for CKD in the NICU), it is difficult to ascertain the broad generalizability of these findings. However, we attempted to recruit caregivers of various ages and backgrounds, at varying time periods out from their child’s NICU stay (eg, 6 months post-NICU discharge vs 2 years post-NICU discharge) in order to improve generalizability to our larger population. Second, the design methods used are novel in health-related research, but they have been well-established in service and product design. Finally, the subjects we recruited were a convenience sample of nonconsecutive caregivers seen at our pediatric nephrology clinic who were willing to participate in research and may not accurately represent a random sample of our patient population.

Despite these limitations, this study represents an important first step in improving communication about kidney health to caregivers and families of those at high risk of kidney disease. The next step in this project is to further develop this communication tool based on caregiver and clinician guidance and to implement the tool in the NICU. Based on the above results and guidance from participants in this project, we are developing a website for family-centered kidney health information and plan to continue to gather input from caregivers to better understand the best ways to present and organize information, how to provide real-world experience and perspectives, and what information caregivers want at specific times during and after their child’s NICU admission. Caregivers of infants admitted to the NICU will be given access to the revised communication tool developed in this study. We will then further assess the impact of the communication tool on their understanding of kidney health, the risk of long-term kidney disease, and follow-up patterns.

## Appendix

Multimedia Appendix 1 Process of human-centered design process to create family-centered communication tool.

Multimedia Appendix 2 Parent comment on barriers and drivers of kidney monitoring and communication strategies.

Multimedia Appendix 3 Preliminary prototypes developed.

## Abbreviations

AKI: acute kidney injury
CKD: chronic kidney disease
NICU: neonatal intensive care unit
