# Supplementary figures and images for: Development of a Family-Centered Communication Tool for Kidney Health in Premature Infants: Qualitative Focus Group Study Using Human-Centered Design Methodology
Source: J Particip Med. 2023 Jul 10;15:e45316. doi: 10.2196/45316 (PMC10366965; doi:10.2196/45316)

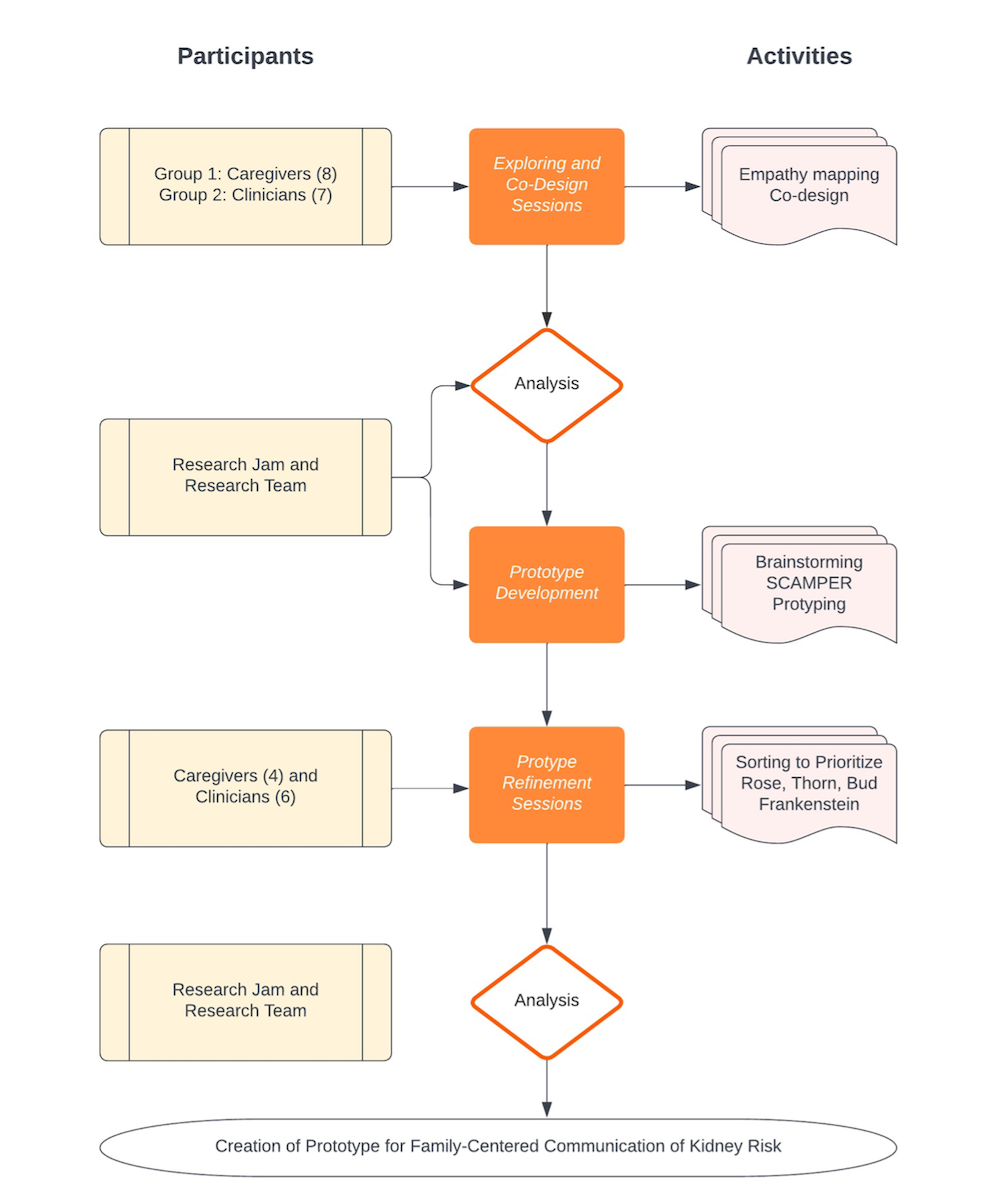

Supplement: Multimedia Appendix 1 [file jopm_v15i1e45316_app1.png]

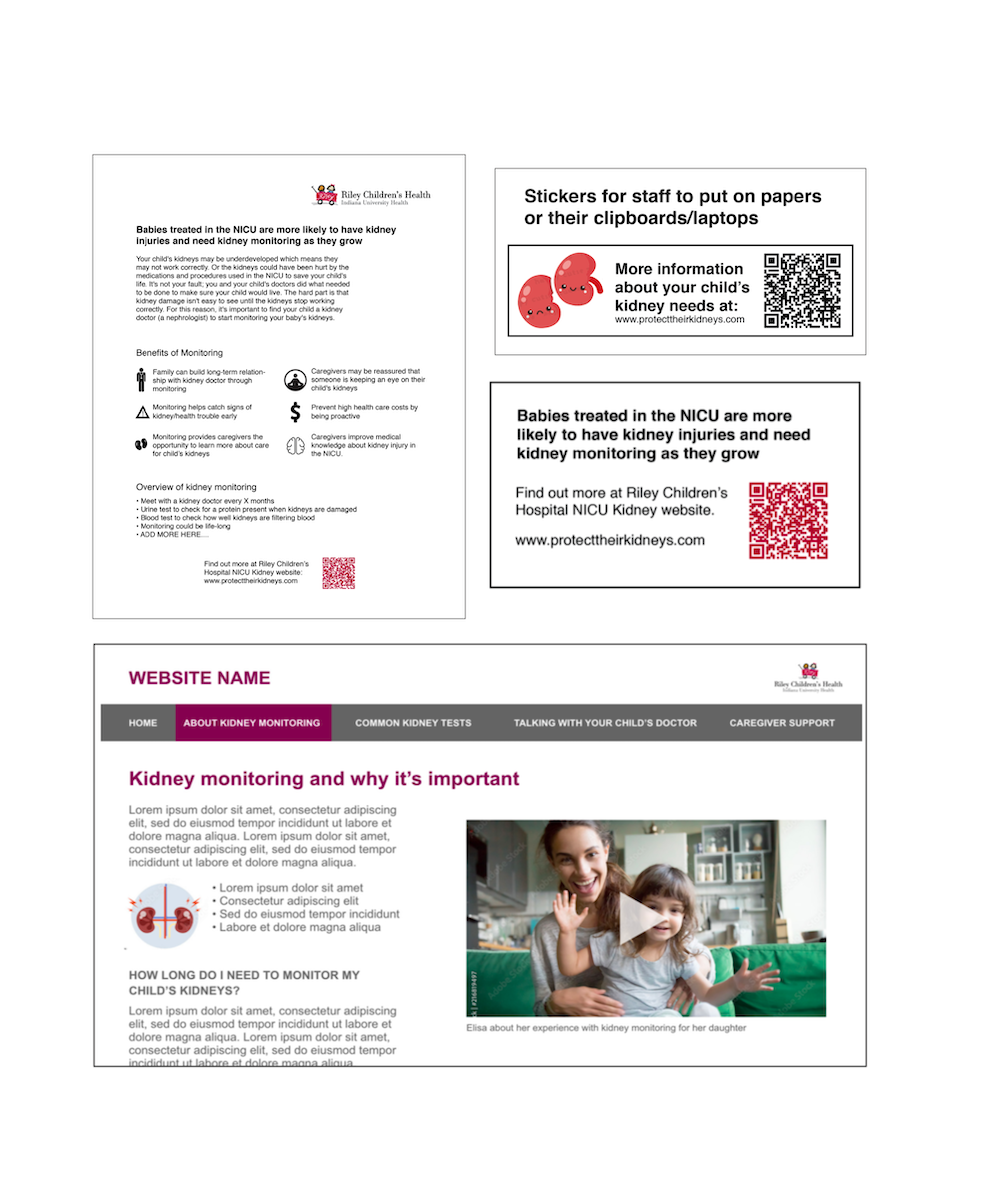

Supplement: Multimedia Appendix 3 [file jopm_v15i1e45316_app3.png]
