# Supplementary material for: Development of a Family-Centered Communication Tool for Kidney Health in Premature Infants: Qualitative Focus Group Study Using Human-Centered Design Methodology
Source: J Particip Med. 2023 Jul 10;15:e45316. doi: 10.2196/45316 (PMC10366965; doi:10.2196/45316)
Supplement: Multimedia Appendix 2 [file jopm_v15i1e45316_app2.docx]

Table S1. Parent comment on barriers and drivers of kidney monitoring and communication strategies.

| **Category** | **Theme** | **Exemplary comments and caregiver quotes** |
| --- | --- | --- |
| Initial NICU Course and Kidney Injury | Difficult and challenging chaotic environment | *Experimental & invasive treatment is an option, no time for questions.*  *Didn’t go over what was really going on or explaining about the medications putting her at higher risk.*  *Thinking now, why didn’t I ask more questions?*  *Wasn’t able to formulate questions in the moment. Wasn’t fully present in the moment. I didn’t totally understand what it meant at the time.*  *Parents are often hearing but not really listening* |
| Barriers to monitoring | Poor cross-specialty coordination | *Visits not well coordinated with other visits.* |
|  | Too many medical appointments and difficult to collect samples | *Appointments when child is in school.*  *It’s hard to draw blood from a baby.*  *Our new pediatrician didn’t think monitoring was needed. Many pediatricians don’t have awareness of the need for kidney monitoring.* |
|  | Poor access to subspecialty providers | *Kidney doctor is not convenient. Doesn’t work with their schedule.* |
|  | Patients age out of pediatrician | *The child seems healthy. They age out of pediatrics and may not stick to it.* |
| Drivers of Monitoring | Plans for monitoring can identify problems early | *You know what you’re dealing with and can make a plan. Helped with some of the worry, good or bad we had answers. Catch something early to prevent transplant. Helpful to know kidney is being looked after.* |
|  | Prevent high healthcare costs by being proactive | *Catch things early to treat. Rich understanding of the patient’s history.* |
|  | Opportunities to learn more about kidney health and improve knowledge | *Learned about things that were not okay for kidneys.*  *Teaching moments about what we should and shouldn’t give for at home meds.* |
| Effective Communication | Reassuring to hear good news | *Reassuring to hear positive things from providers.* |
|  | Providers worked with caregiver to help them understand topics | *Nurses and doctors were more than happy to explain. Nurses spoke really well - dumbed it down for us.* |
|  | Conversations between caregivers and providers occurred privately | *Happens in a separate, private room.* |
| Ineffective Communication  ` | Not told monitoring was needed until discharge or follow-up or were not told monitoring might be life-long | *No one said kidney info until discharge. Got better explanation at follow up. Told we’d need follow-up but weren’t told it’d be life-long* |
|  | Conversations felt rushed and sporadic, not holistic | *Conversation about monitoring felt rushed. Conversations felt piecemeal. Hard to put pieces together.* |
|  | Conversation location not conducive to serious talk | *The location of our conversation wasn’t private.*  *Conversation may happen in open area without privacy.* |
